# Rehabilitative strategies after filtering procedure in glaucoma

**DOI:** 10.1038/s41598-022-20191-x

**Published:** 2022-10-07

**Authors:** Enzo Maria Vingolo, Lorenzo Casillo, Giulia Mecarelli, Paolo Giuseppe Limoli

**Affiliations:** 1 Department of Sense Organs: University “Sapienza” of Rome “Polo Pontino” Ophthalmology Unit “A. Fiorini” Hospital, Terracina, Italy; 2 Centro Studi Ipovisione, Milan, Italy

**Keywords:** Medical imaging, Quality of life

## Abstract

Glaucoma is one of the leading causes of non-reversible blindness worldwide, and almost 6 million people are estimated to be impaired visually in advanced stage of glaucoma. Recently, several studies on glaucoma has been focused towards new therapeutic approaches based on mechanisms independent from IOP control. Effects of new therapeutic agents, visual psychophysical training, or complementary medications targeting optic pathways today seem to be a relevant and effervescent field of research. The goal of the study is to evaluate in glaucoma patients if a rehabilitative strategy with a biofeedback training with microperimetry may be useful after surgery in recovery visual performance even when visual field defects are present in IOP is well controlled environment. Were enrolled 24 patients (28 eyes) with Primary Open Angle Glaucoma (POAG) (mean 63 range: 49–75 years) from our Glaucoma Center after filtering surgery. All patients after one months from surgical intervention underwent to a complete ophthalmologic examination: IOP measurement, gonioscopy, visual field and SD-OCT at baseline of RNFL thickness. In some cases, were included in the study both eyes because in POAG frequently clinical conditions are different in each eye, and secondarily new fixation target retinal location (TRL) was chosen based on single eye retinal sensitivity. Best corrected visual acuity was significantly increased after the training from 0.61 to 0.479 (p = 0.00058) with no change in refractive error. After the biofeedback patients presented increased value in Mean retinal sensitivity from 14.91 to 15.96 (p = 0.0078).Fixation stabilitywas improved either according to Fuji classification (increased from 75.1 to 81.3% p = 0.0073) or BCEA value, reduced from 8.7 to 6.0 square degrees (p = 0.013) we noted a marked increase in this parameter with better performances and satisfaction by the patient. RFNL thickness: no change was noted (p = 0.505) in this value as an indicator of disease’s stability. Our data indicate that MP-3 Biofeedback may be a good strategy to reduce the impairment of the Glaucoma Patient.

## Introduction

Glaucoma is one of the leading causes worldwide of non-reversible blindness, (5.7 million people are estimated to present severe visual loss in final stage of glaucoma)^1^. First risk factor in glaucoma it is high levels of intraocular pressure (IOP)^2^; even, more recent studies pointed out that also non-IOP-dependent mechanisms play a role on the pathogenesis and progression of glaucoma, and between most relevant we may include vascular dysregulation in optic nerve head perfusion^3^, high amount of free reactive oxygen species^4^, moreover also excitotoxicity^5^ and autoimmunity^6^, are mechanisms that can suggest why glaucomatous optic neuropathy may occur even with normal values of IOP.

Nowadays main treatment of glaucoma eyes is focused on IOP reduction with different drugs, that significantly delays glaucoma progression, optic nerve head damage and visual field loss^7^. Ophthalmological procedures in late evolutionary phase, usually achieve the goal in almost all patients, based on intensive treatment with anti-glaucoma drugs, that needs rigid patient compliance, or more invasive strategies as laser treatment (either thermic, micro pulsed or selective), and surgical intervention as filtering procedures or mini-invasive glaucoma surgery (MIGS).

In advanced phase glaucoma patients usually show IOP control relatively stable and often in normal range, with severe Visual Field constriction and mild or moderate visual loss configuring low vision. Consequences of these clinical conditions are that glaucoma patients have a very poor quality of sight and life.

As an attempt to relieve this situation, several researchers in the field of glaucoma have been recently focused on new therapeutic approaches based on mechanisms independent from IOP control. The effect of these novel therapeutic approach^8–10^ including visual psychophysical exercises^11^, and complementary medications^12–14^ targeting visual pathways wellness are very promising and subject of attention.

Some ophthalmologists point their interest on this complementary or alternative medicine (CAM), supporting visual performances in glaucoma patients, with the advantage of targeting at same time IOP-dependent and non-IOP-dependent damage mechanism^15,16^.

Studies showed that one in every nine patients with glaucoma uses CAM for the support in the treatment of this disease^17^. This study was based on a wide range of CAM categories, (e.g. herbs, dietary support and visual performance training practices).

Since the introduction of a new technical device for fundus related perimetry (microperimetry MP) in the 2 k century a new era was open for CAM in several diseases as described in several studies^18–25^. Microperimetry has the advantage of a repeatable strategy for analyzing the psychophysical responses, and new feature a biofeedback strategy for visual training with the scope of increase visual performances in normal and pathologic eyes.

Microperimetric Biofeedback was proposed in 2007^18^ and successfully proposed by our and other groups worldwide in several report on retinal disease, but this technique rarely was used in Glaucoma patients because it is very efficient in low vision due to central scotoma when the patient loses fixation, and targeting strategies are easily reached by the patient. Unfortunately, this situation in glaucoma patients usually happens only in late stage of the disease in which there is a complex damage of central retinal nerve fibers and consequently severe visual field constriction and central visual loss. Glaucoma filtering surgery usually determines a drop in visual performances that often it is hard to recover^26^ nevertheless an adequate IOP control and conventional treatments seem not to influence this course.

The goal of our study is to point out if in glaucoma patients after surgery, when visual field defects are present and amplified by the procedure and recovery in visual function is slow, biofeedback training with microperimetry may be useful. Our primary endpoint was the evaluation of fixation Behavior Contour Ellipse Area (BCEA), and improvement of this is considered with a resulting minor area in which 95% of fixation points fall, secondary endpoint was the improvement in retinal sensitivity and visual acuity in presence of a glaucoma stable clinical condition evaluated by the mean of RFNL thickness at OCT.

## Materials and methods

A total of 24 patients (28 eyes) with late stage of Primary Open Angle Glaucoma (POAG) (mean 63 range: 49–75 years) were enrolled from Glaucoma Centre in Terracina University Hospital from January 2014 to December 2019 after filtering procedure (trabeculectomy according to Cairns). These patients were randomly selected and progressively numbered as they underwent to surgery, we used the online randomization (http://www.graphpad.com/quickcalcs/index.cfm) selecting random numbers and then assigning subjects to groups to undergo our observational study.

Reason for the decision to include also bilateral involvement was made because even if glaucoma has a bilateral inherited situation, clinical conditions may be different between two eyes depending from several factors. Glaucoma onset may be different, response to treatment, visual field and optic nerve head status may have different evolutionary status. In this view we decided to consider also bilateral involvement as singular cases in 4 patients.

Before enrolment written informed consent was given by each participant of the study. Institutional board approval was obtained from Ethics Committee of our Department Of Ophthalmology University Sapienza in Rome (Determina 127 del D. 14.9.2020). Tenets of Declaration of Helsinki were observed in all procedures.

1. Eligibility criteria included: Filtering procedure more than 8 weeks before when most of beneficial effects of surgical procedures were already achieved, clinical features of POAG with compromised Visual Field, corrected visual acuity better than 20/200, normal lifestyle based on Mediterranean diet, mild to moderate physical exercise, abstinence of alcohol and tobacco products, no systemic hypertension or dyslipidaemia, and no excessive exposure to the light or ultraviolet rays.
2. Exclusion criteria were patients with secondary ocular Hypertension, uncollaborative, we have also excluded subjects with other ocular pathologies, severe dioptric media opacities, patients who had a history of intravitreal injection therapies and previous retinal laser treatment, and those with a history of intraocular or vitreoretinal surgery within 6-month.

All patients underwent a complete ophthalmologic examination IOP measurement, gonioscopy, visual field and SD-OCT at baseline of RNFL thickness. In some cases, both eyes were considered for the study because clinical conditions were different in each eye.

Microperimetry was performed with the Microperimeter MP3 (Nidek Technologies) in all subjects. A red circle of 1 diameter was used as fixation target, 31.4 apostilbs white background illumination, Goldmann III stimuli with a projection time of 200 ms, in a grid, centered on the fovea, of customized 33 stimuli around 10° (10–2 grid). An initial projecting sensibility of 8 dB and a 4–2 staircase strategy were used. Auto-tracking and auto-alignment functions were turned on to achieve maximal accuracy of measurements. Was automatically analyzed the percentage of fixation points located into 4° centered on the fixation point (according Fiji 2002). The fixation pattern, stability, and fixation zone were classified in percentage of fixation points within 2° central degrees according to Fuji and associates (27) and classification of BCEA that better fit in our view for a statistical analysis. “Pretest” option was selected to avoid learning effect and spherical error was considered before starting the examination. MP-3 microperimetry was performed at baseline and at the end of visual rehabilitation protocol (i.e., after 10 weeks). Data on retinal sensitivity and fixation stability were collected at the end of each session. Bivariate contour ellipse area (BCEA, deg^2^) and Fiji classification Fixation stability were reported. The BCEA was normalized by logarithmic transformation for statistical analysis (Shapiro–Wilk test).

Two Microperimetric indexes were considered to evaluate effectiveness of treatment with acoustic biofeedback, Mean retinal sensitivity and Fixation Stability (Fuji classification and BCEA extension):

Biofeedback strategy was performed as described in our previously presented report^1,2^.

1. Based on baseline Microperimetric report, new fixation target retinal location (TRL) was chosen on single eye retinal sensitivity map allowing upper 5 better consecutive point zone.
2. A 1-degree red cross was used as fixation target of visual rehabilitation training that was placed on TRL.
3. The session (10 consecutive minutes) was started stimulating the patient to fix the cross, with acoustic biofeedback (higher sound when actual fixation is nearest to TRL) and inviting him to maintain high level of sound.
4. Each time that the patient loses the fixation the operator stimulates the patient to reach again the TRL.
5. At the end of the session (considering bilateral facilitation pathways at retinal, geniculate, and cortical levels), was always stimulated the fellow eye.
6. In each session report was included the record of fixation evolution during the elapsed time.
7. Rehabilitative protocol, using the MP-3 acoustic target biofeedback, consisted in 10 training sessions of 10 min for each eye, these sessions were repeated once a week.

Best Correct Visual Acuity (BCVA) was recorded at baseline and after 10 weeks; was measured using a standard EDTRS visual acuity chart (CSO electronic chart, Firenze, Italy) and then in logarithm of the minimum angle of resolution (logMAR) for statistical analyses.

RFNL and sdOCT scans were taken with Heidelberg Spectralis OCT (Heidelberg Engineering GmbH Germany). We have chosen to include RNFL analysis that has a long term value of IOP control. Statistical analysis was performed using Student’s-test and values less than 0.05 were considered statistically significant. For statistical analysis, we used SPSS (Chicago, IL, USA) v 14.0.2. for Windows.

### Ethics approval and consent to participation

Ethics approval and consent to participate from our Institutional board was obtained from ethics committee of Department of Sense Organs University Sapienza of Rome (O.d.S D. 14.9.2020). Tenets of Declaration of Helsinki were observed in all procedures, informed consent was obtained from all participants before including them in the session study.

## Results

### Best corrected visual acuity

**Figure 1:**
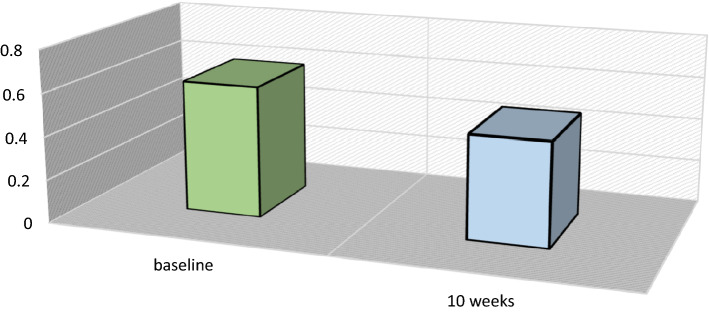
BCVA changes after biofeedback.

after the training visual acuity was significantly improved from 0.61 LogMAR to 0.479 LogMAR (p = 0.00058) with no change in refractive correction, 6 eyes presented an increase more or equal to two lines after the training and only one denoted a reduction of one line in visual acuity. (Fig. 1).

### Microperimetric indexes

**Figure 2:**
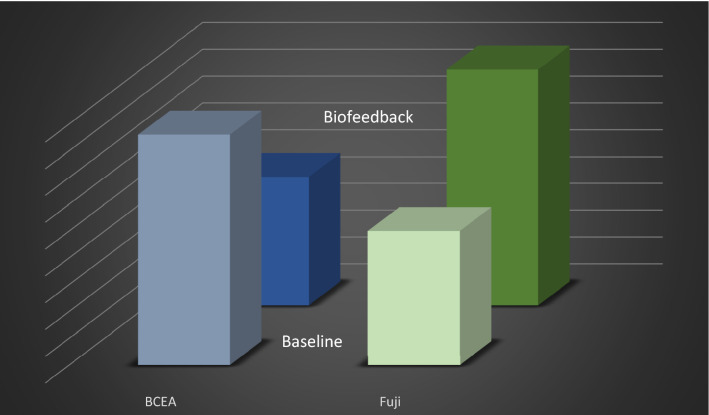
Fixation Stability (BCEA and Fuji %) after biofeedback.

1. Mean Retinal Sensitivity: after the biofeedback patients presented increased value from 14.91 to 15.96 dB (p = 0.0078) this value indicates that retina has improved the ability to recognize lower luminance stimuli.
2. Fixation stability: Meaning of this value is that macula has gained ability in lock-in fixation target and to maintain fovea on this point during the exercise time. Either according to Fuji classification (increased from 75.1 to 81.3% p = 0.0073) or BCEA value, in this case minor value of the area results in less wide fixation saccades, reduced from 8.7 to 6.0 square degrees (p = 0.013) we noted a marked increase in this parameter with better performances and satisfaction by the patient (Fig. 2) at baseline and at the end of the rehabilitative training.

### RFNL thickness mean value (Fig. 3)

**Figure 3:**
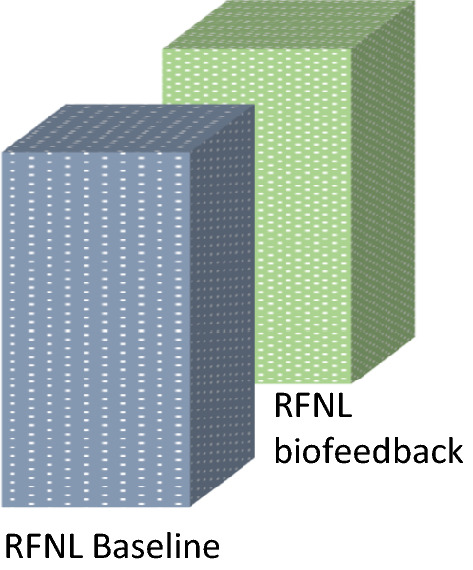
RFNL thickness.

Invariance of this morphologic parameter after the observation time during the biofeedback in which no change was noted (p = 0.505) suggested us this value as a stability indicator of the disease.

## Discussion

Glaucoma, as stated before, in the world is a leading cause of acquired blindness, in most advanced cases, presence of scotoma involving the fixation area usually becomes relevant. Visual function in glaucoma patients is usually evaluated with basic visual acuity and static computerized perimetry with dedicated strategy. Both tests in our opinion, in late stages of the disease, are incomplete to investigate aspects of macular function, visual field, because the location and number of points, might not be adequate to detect small scotoma located around the central foveolar area and in advanced glaucoma unstable fixation affects reliability of test results. In late glaucoma stage a deep loss of retinal sensitivity produces significant impairment and functional symptoms that step into low vision area ad have as a further evolution blindness. Our preliminary study is focused on the possibility that a complementary or alternative medicine (CAM) may add a functional benefit to a common care treatment of glaucoma patients.

Microperimetry and rehabilitative tools was already used in literature with the aim to diagnose and improve fixation stability in patients with several retinal diseases. In advanced glaucoma, may be possible obtain good results with MP-3 microperimetric biofeedback, with the goal to activate and train new PRL to improve several functional parameters as: fixation stability, BCVA, reading speed and consequently quality of life. Investigating fixation patterns in glaucomatous patients Takanori et al.^16^ using the MP-1 microperimeter in 39 eyes with advanced glaucoma concluded that was possible individuate specific fixation patterns and scotoma maps of retinal sensitivity in eyes with glaucoma with a strict correlation with optic nerve head conditions.

Techniques of Biofeedback were applied to vision^27^ and still now, considering methodological and physiological aspects, are investigated by several authors^19,20,28^. Different visual rehabilitation devices that use different strategies of fixation are proposed for biofeedback purpose. These devices were developed in the past century like “Accommotrack Vision Trainer” (The Nasa Connection, Seattle, WA) a very basic instrument that allowed low level fixation control and few programs dedicated to low vision patients. More complex devices were developed in the late nineties and early 2000, these instruments were characterized by integrated high level of eye tracking with biofeedback system fundus related MP-1 microperimeter (NIDEK Technologies Srl, Padua, Italy), or the MAIA2 (Centervue Padua Italy). Others were based on electrophysiological control of fixation obtained by steady state VEP (visual trainer LACE instruments).

Biofeedback training used in different diseases, was supposed obtain better visual performances facilitating neural transmission involving intraretinal neurons and interneurons connections between the retina and brain, supporting a “remapping phenomenon” as described from several Authors^21,22^. Andrade et al.^29^ these Authors demonstrated that glaucoma patients in their vision, frequently do not have perception of the scotoma, because, in case of optic pathway damage, cortical receptive fields originating from this region are not inactive but show more sensitivity to stimuli localized in surrounding areas, by the mean of connections driven by horizontal cells in the inner layers of the retina, geniculate area and cortex.

Two distinct steps, characterize this phenomenon with different time scale: (a) a redistribution of receptive fields (RFs) in the area of the lesion, and (b) a progressive enlargement of neuronal fibers projecting in areas surrounding the scotoma, resulting in a new visual field configuration. Although the gradual rearrangement mechanisms are becoming more evident in the literature, several authors underline visual pathways rearrangement during the training, driven by an increase in synapses number, faster interneurons mediators and neurotransmitter reuptake, the trigger and first step of this process remains still unclear.

Position of glaucoma lesions projected in the retinal area as corresponding to the scotoma, send some kind of activity, from the undamaged neurons, to cortical surrounding neurons located in the area the lesion^29^. The brain is able to modify itself, for intrinsic cortical plasticity, to adapt to new background modifications depending to damage of the neural system. This author also underlies the learning and attention processes involved in this rearrangement. Safran and Landis^30^ stated that, “Cortical changes occurring after focal visual differentiation modify visual perception by filling in visual field defects with information from the area surrounding the scotoma”.

These processes cause, in glaucoma subjects, tendency to underestimate their defects, distortion in spatial perception, in visual field amplitude or in cortical plasticity. Sum of effects can delay patient’s detection and identification of visual field defects, and consequently the treatment, meanwhile also affecting perception and outcome of some diagnostic procedures^30^.

Auditory biofeedback, as found by Mezawa et al.^27^, were applied in treatment congenital nystagmus, patients reported at the end of training, a subjective sensation of better vision and improvement of foveation time, VEP amplitude, and threshold spatial frequency. Visual training effects of auditory biofeedback have also been studied in myopia to improve visual acuity^4,25,31^. Another technique based on fundus related perimetry (microperimetry) with Scanning laser ophthalmoscope (SLO) provides functional results by direct visualization of the macular area.

Results of this method underline a correspondence point-to-point between fundus images and retinal sensitivity threshold. Instability of fixation, usually observed in static computerized perimetry, is a possible misleading factor that can result in difficult to interpreter findings, especially in eyes with low visual acuity or blindness. SLO microperimetry allows an accurate, direct and on line visualization of the stimuli when and where they are presented on the retinal surface, with a very short distance between two single stimulated points (less than 30’ instead 3° of standard computerized perimetry): this ultimately allows high accuracy in fixation monitoring and correlation directly between anatomo-pathological features and retinal function^32^.

Rudimentary techniques of auditory Biofeedback were originally used for the treatment of different forms of ametropia (myopia, astigmatism, and presbyopia), nystagmus and amblyopia^33,34^.

In our previously presented studies^28,35^ we have underlined that in rehabilitation strategies of low-vision patients biofeedback with MP-1 is really efficient in patients with different diseases involving the posterior pole (age-related macular degeneration, vitelliform dystrophy, Stargardt’s disease, myopic macular degeneration, post-traumatic macular scar, cone dystrophy) reporting improvements visual performances (visual acuity, fixation behavior, retinal sensitivity and reading speed) and confirmed by Pacella et al.^23^.

Visual biofeedback training using the Visual Pathfinder (LACE Inc., Rome) was investigated by other authors in patients with high myopia evaluating VEP output, this study demonstrated improvements in visual performances (BCVA, fixation stability and retinal mean sensitivity, amplitude of the main peak of the pattern VEP). This functional training improved visual performances and consequently better quality of life, with a positive psychological effect on these patients that frequently present a depressive trait^24^.

Patients with retinitis pigmentosa were also treated with MP-3 and Visual Pathfinder rehabilitation, and in this case was shown that a stimulus with flickering pattern can increase visual acuity and VEP amplitude more than plain luminance stimulus, probably receptive field stimulated by the alternance of black and white checkerboard were potentiated, allowing an easier rearrangement of signaling originating from the surviving retinal rod-photoreceptors^36^.

As more elaborated Auditory biofeedback, instead driven by a luminous target pointed on a training retinal location (TRL), by a pattern stimulus (a flickering black and white checkerboard activated when patient’s fixation is pointed on a defined TRL) were evaluated in age-related macular degeneration patients^4^. Both groups, in comparison with the baseline, showed better visual performance after rehabilitation but the biofeedback with pattern flickering stimulus was found to be significantly better and faster in training the patients to reach their PRL compared with the standard one. In the Authors opinion this suggests that, in the damaged retina, neuronal plasticity might override dead photoreceptor and outer retinal layers involving residual inner retina layers rich in surviving cells, to obtain signal amplification and integration at retinal and/or cortical level.

After visual training techniques improvement mechanisms of visual function may be controversial and various hypotheses can be advanced, in our opinion one of the most relevant is better coordination in oculomotor control and “searching capacity”.

Use and improvements in eccentric fixation due to the conditioned reflexes determined from the auditory peak could also be a mechanism^37^. In our opinion, it is very relevant consideration that the improvement in visual function, mainly depending from foveation time, could be obtained after the training as a result of better ability for the patients to manage their residual visual function and reach or maximize full potential.

Residual vision activation theory was suggested by Sabel et al.^38^ to explain in which way the system may reactivate or restore visual function. In early 2000 was proposed a new technique: vision restoration therapy (VRT) as complementary treatment for AMD or Diabetic Macular edema; VRT involves a specific pattern of visual stimulation, directed at the border of the scotoma and the blind field, training this seen/not seen search may finally result in expansion of visual fields in individuals with brain or optic nerve injury as reported by Kasten and Sabel^39^ (Kasten et al. 1998). Romano et al.^40^. These papers demonstrated improvement in detection of stimuli and BCVA with VRT daily sessions. Measuring with suprathreshold visual field testing, resulted a shift of the position of the border of the blind field. So that the authors conclude that VRT is a useful intervention for rehabilitation in some patients with visual field defects from retro-chiasmatic ischemic lesions.

Restore vision and reduction of the scotoma extension can be achieved, but these results are related to the residual tissues and their activation state. Training unfortunately does not lead to permanent changes, interrupting biofeedback causes slow regression in performances, maintaining these levels of visual functions, require repetitive stimulation, possibly over days weeks or months in relationship to the deepness of the defect.

Changes in visual performances are partly determined by psychophysical and subjective variables as learning effect, motivation, level of attention, psycho-physical capacities, type of environment and influence of the examiner^41^ so there is a need of high compliance by the patient.

Age, as frequently reported in the literature^42–44^, is the major risk factor for glaucoma because degeneration of the retinal ganglion cells (RGCs) and its consequence of optic disk cupping characterizes all patients as clinical finding and this phenomenon has been compared to the visual impairment seen in patients with Alzheimer’s disease in which very often is associated undiagnosed findings of glaucoma. Also RGC death or optic nerve fiber degeneration may be present as part of biological mechanism very similar in most degenerative diseases of central nervous system^45^. New findings in recent paper have proven glaucoma-like axonal damage until the lateral geniculate nucleus and visual cortex from can^46^. This suggest, in our opinion, that glaucoma could be responsible of reduced visual performances as single visual field defects in patients affected by dementia moreover this thought suggests that developing an integrated approach with an interdisciplinary involvement, may lead to discoveries at different levels and underlines how favoring the connections between different specialties push forward the research^47^.

## Conclusions

Our study focused only on complementary or alternative medicine (CAM) has the main target to evaluate if these techniques may be applied to a selected population independently from the clinical conditions.

Certainly would be interesting evaluate which clinical condition of glaucoma patients (evaluating Visual Acuity or Visual Field) has a better chance to reach easily better condition but in this study we have done the choice to evaluate all patients independently from their clinical condition, otherwise, we need to stratify the study group for clinical conditions (VA and VF) type of glaucoma surgery (MIGS, Stents or filtering procedures) and we may have the need to enlarge the sample size to more than one hundred studied subjects. This certainly will be the next step of our group and we need to involve more Glaucoma Centers and surgeons in different hospitals.

Results of this study demonstrate that biofeedback rehabilitation with MP-3 has marked influence on patients with advanced glaucoma restoring better visual performances and satisfactory results in terms of reading capabilities and subjective perception in daily activities. All investigated glaucoma patients showed better fixation and more precise retinal sensitivity and the results of these increased performances was the improvement in reading speed and visual acuity. BCEA in our study revealed itself as a very affordable parameter to monitor visual function in advanced stages of glaucoma where BCVA is too low and sensitive to monitor small changes. In our opinion BCEA is a very affordable and easy to obtain parameter for monitor fixation stability, as logMAR evaluation smaller values indicate that fixation is more stable^48^.

Low-vision stimulation and rehabilitative techniques in our experience suggests that in advanced glaucoma could be possible to improve residual visual function restoring better visual performances determining a better quality of vision with positive relapses on psychological situation. Larger numbers of patients may be request for further studies that may allow us to test different strategies dedicated to point out more efficient new PRL in each patient.

## Data Availability

Data and material will be available at AUSL Latina “A. Fiorini” Hospital 04120 Terracina Italy. The datasets used and/or analysed during the current study available from the corresponding author on request.

## Abbreviations

IOP: Intraocular pressure
MIGS: Micro Incisional glaucoma surgery
CAM: Complementary or alternative medicine
MP: Microperimetry
BCEA: Behavior contour ellipse area
RFNL: Retinal nerve fiber layer
OCT: Optical coherence tomography
POAG: Primary open angle glaucoma
SD-OCT: Spectral domain optical coherence tomography
TRL: Target retinal location
LogMAR: Logarithm of minimal angle of resolution
PRL: Preferential retinal location
BCVA: Best correct visual acuity
VEP: Visual evoked potentials
SLO: Scanning laser ophthalmoscope
VRT: Vision restoration therapy
